# Is metabolic syndrome a modifiable risk factor for gout?

**Published:** 2022-12

**NOVEMBER 23, 2022** - In a study published in Arthritis & Rheumatology that included nearly 1.3 million men aged 20–39 years who participated in three serial health check-ups at two-year intervals, men with metabolic syndrome (MetS) and those who developed MetS—especially those with the MetS components of elevated triglycerides and abdominal obesity—had higher risks of developing gout.

Among participants, 18,473 developed gout, and those with MetS at all checkups had a nearly four-fold higher risk than participants who were MetS-free. Development of MetS more than doubled the risk of incident gout, whereas recovery from MetS reduced incident gout risk by nearly half.

“This is the first large-scale study to explore the association between dynamic changes in MetS and risk of gout,” said co–corresponding author Jaejoon Lee, MD, PhD of the Sungkyunkwan University School of Medicine, in South Korea. “Prevention and recovery from MetS can significantly lower the risk of gout in young adults.”

***URL upon publication: https://onlinelibrary.wiley.com/doi/10.1002/art.42381***

***Full citation:** “Altered Risk of Incident Gout According to Changes in Metabolic Syndrome Status: A Nationwide Population-Based Cohort Study of 1.29 Million Young Men.” Yeonghee Eun, Kyungdo Han, Seung Woo Lee, Kyunga Kim, Seonyoung Kang, Seulkee Lee, Hoon-Suk Cha, Eun-Mi Koh, Hyungjin Kim, Jaejoon Lee. Arthritis Rheumatol; Published Online: 22 November 2022 (DOI: 10.1002/art.42381)*.

*Copyright © 2019 The Cochrane Collaboration. Published by John Wiley & Sons, Ltd., reproduced with permission*.

